# Neural Network Model of Vestibular Nuclei Reaction to Onset of Vestibular Prosthetic Stimulation

**DOI:** 10.3389/fbioe.2016.00034

**Published:** 2016-04-20

**Authors:** Jack DiGiovanna, T. A. K. Nguyen, Nils Guinand, Angelica Pérez-Fornos, Silvestro Micera

**Affiliations:** ^1^Center for Neuroprosthetics, Bertarelli Foundation Chair in Translational Neuroengineering, École Polytechnique Fédérale de Lausanne, Lausanne, Switzerland; ^2^Cochlear Implant Center for French Speaking Switzerland, Service of Otorhinolaryngology – Head and Neck Surgery, Geneva University Hospitals, Geneva, Switzerland

**Keywords:** vestibular prosthesis, electrical stimulation, functional models, adaptation, physiological, synapses, vestibular ocular reflex

## Abstract

The vestibular system incorporates multiple sensory pathways to provide crucial information about head and body motion. Damage to the semicircular canals, the peripheral vestibular organs that sense rotational velocities of the head, can severely degrade the ability to perform activities of daily life. Vestibular prosthetics address this problem by using stimulating electrodes that can trigger primary vestibular afferents to modulate their firing rates, thus encoding head movement. These prostheses have been demonstrated chronically in multiple animal models and acutely tested in short-duration trials within the clinic in humans. However, mainly, due to limited opportunities to fully characterize stimulation parameters, there is a lack of understanding of “optimal” stimulation configurations for humans. Here, we model possible adaptive plasticity in the vestibular pathway. Specifically, this model highlights the influence of adaptation of synaptic strengths and offsets in the vestibular nuclei to compensate for the initial activation of the prosthetic. By changing the synaptic strengths, the model is able to replicate the clinical observation that erroneous eye movements are attenuated within 30 minutes without any change to the prosthetic stimulation rate. Although our model was only built to match this time point, we further examined how it affected subsequent pulse rate modulation (PRM) and pulse amplitude modulation (PAM). PAM was more effective than PRM for nearly all stimulation configurations during these acute tests. Two non-intuitive relationships highlighted by our model explain this performance discrepancy. Specifically, the attenuation of synaptic strengths for afferents stimulated during baseline adaptation and the discontinuity between baseline and residual firing rates both disproportionally boost PAM. Comodulation of pulse rate and amplitude has been experimentally shown to induce both excitatory and inhibitory eye movements even at high baseline stimulation rates. We also modeled comodulation and found synergistic combinations of stimulation parameters to achieve equivalent output to only amplitude modulation. This may be an important strategy to reduce current spread and misalignment. The model outputs reflected observed trends in clinical testing and aspects of existing vestibular prosthetic literature. Importantly, the model provided insight to efficiently explore the stimulation parameter space, which was helpful, given limited available patient time.

## Introduction

Vestibular prosthetics are designed to restore sensory information in chronic and severe loss of natural vestibular organ function. Overall, the number of persons with reduced vestibular function is estimated to be about 35% (Agrawal et al., 2009). In particular, vestibular loss can be debilitating in cases of severe bilateral vestibular loss (BVL). In these cases, adaptive mechanisms such as central compensation fail to improve the patient’s condition, despite intensive physical therapy (Zingler et al., 2007). Patients are both restricted in activities of daily life (Guinand et al., 2012) and have a poor outlook on recovery (Sun et al., 2014). This highlights the urgent need for new treatment options (Van De Berg et al., 2011).

One treatment option currently in the prototype stage is vestibular prosthetics. These prosthetics are designed to mimic the afferent firing rates that would be generated by healthy semicircular canals (SCCs). A configuration that has been used both in animal models (Gong and Merfeld, 2002; Della Santina et al., 2007) and humans (Van De Berg et al., 2011; Perez Fornos et al., 2014) achieves this via monopolar stimulation from electrodes in close proximity to the ampullae or vestibular afferents. This electrical stimulation engages afferents from the SCCs and establishes a clear relationship between the delivered simulation and sensory response, and this relationship is possible due to the homogeneous organization of these sensory organs. In contrast, the peripheral vestibular organs that sense linear acceleration (the otoliths) have a heterogeneous organization (Lewis, 2015). Prosthetic designers have thus far only attempted to restore canal function and not otolithic function because of (i) the relatively simple organization (and accessibility) of SCC afferents and (ii) the complex anatomo-physiology of the otoliths. Given these constraints, current prototypes aim to restore rotational, but not translational, information.

In a healthy animal, changes in firing rate of SCC afferents correlate well with the rotational velocities around axes aligned with that canal (Fernandez and Goldberg, 1971). Modulating electrical stimulation parameters, e.g., pulse rate or pulse amplitude, applied to these afferents can similarly induce corresponding eye movements (Suzuki and Cohen, 1964; Merfeld et al., 2007; Davidovics et al., 2012). Detailed finite element models have been developed to simulate recruitment of vestibular afferents based on the applied electrical stimulation; then changes in eye movements can be calculated as a function of the number of afferents recruited (Marianelli et al., 2015). That model predicted symmetrical changes in eye velocities when pulse rate modulation (PRM) was applied, but sharply non-linear changes when pulse amplitude modulation (PAM) was applied. Overall, it well approximated the literature and also highlighted a range of comodulation possibilities (Marianelli et al., 2015), as experimentally demonstrated in Davidovics et al. (2012).

We build on experimental and modeling work discussed in the prior paragraph but study a different time point in prosthetic use. The existing literature evaluates modulation relative to a baseline stimulation rate. Importantly, at the onset of this baseline rate, there is a discontinuity in stimulation from 0 pps (pulses per second) to a baseline rate (e.g., 200–400 pps). Since stimulation can overwrite residual resting firing rate, there is a corresponding discontinuity in afferent activity. This discontinuous and sharp step from resting rate imposes a transient nystagmus that must attenuate before the prosthetic can be used. This strategy is reflected in common experimental paradigms, e.g., “baseline stimulation was applied and after *N* minutes nystagmus attenuated; we then modulated stimulation and measured eye-movement output.” The timeframe *N* is species- and time-dependent [e.g., Merfeld et al. (2006), Guyot et al. (2011), and Davidovics et al. (2012)]. Our focus is on that often-skipped timeframe.

Specifically, could synaptic plasticity between the afferents and the vestibular nuclei accommodate this transient nystagmus attenuation? Arnold and Robinson (1997) built a six-layer neural network composed of four different types of neurons connecting the SCCs with the ocular movements. We model the synaptic strength between each afferent (two types) and the vestibular nuclei as in Arnold and Robinson (1997), instead of a global function between afferent firing and stimulation parameters to eye movements (Marianelli et al., 2015). We start from healthy SCCs with afferents having a distribution of resting firing rates, variances, and response magnitude to rotation. There is a corresponding distribution of synaptic strengths. We then simulate BVL, baseline prosthetic stimulation, and finally pulse modulation. The onset of baseline stimulation is a “perturbation” and the model adapts the synaptic strengths to reduce error (no aberrant eye movements). Any subsequent changes in stimulation, either modulation or a change in baseline rate, will induce eye movements. We examine how synaptic strength adaptation affects subsequent VOR to modulation of stimulation. Specifically, how does adaptation of a portion of the synapses affect the efficacy of subsequent PRM or PAM? Additionally, are there non-obvious characteristics of prosthetic stimulation exposed through this model?

We abstract a simple neural network model from biological principles to model the pathway including the electrode, afferents, vestibular nuclei, and eyes. Model inputs are electrical pulse stimulation amplitude and rate. The model output is eye velocity around a single axis (e.g., horizontal, vertical, or torsional); these three axes comprise the space of eye movements (Haslwanter, 1995). Conceptually, the model could produce velocity around three axes to accommodate current spread or “cross talk.” However, we will discuss in Section “Results” why we restricted our analysis to a single axis.

Simulations of acute (short-duration trials within the clinic in humans) vestibular prosthetic modulation after this adaptation predict eye movements that followed the same trends as acute clinical testing for PAM and PRM^1^. Searching over a spectrum of resting firing rate distributions showed minimal influence on relative stimulation efficacy. Finally, we simulated a comodulation of pulse rate and pulse amplitude that was previously used in animals (Davidovics et al., 2012). The model predicts both boosted outputs for combined stimulation and equipotent outputs using less PAM, an important factor in reducing current spread and misalignment of eye-movement responses.

## Materials and Methods

The model is divided into two main components: electrode–nerve interface and nerve–nuclei interface. The model has one layer of free parameters, specifically the synaptic strengths. These are adapted during the onset of prosthetic stimulation. We describe the model components below. Experiments were performed in accordance with the Declaration of Helsinki and approved by the ethics committees of the University Hospitals Geneva (NAC 11-080).

### Electrode–Nerve Interface

Rotation is normally sensed by hair cells within the ampulla; it results in modulated firing rates of vestibular nerve fibers. In patients with BVL, these cells are significantly less sensitive or non-responsive to rotational stimuli or may be lost. The electrode–nerve interface is built upon existing physics of electrical stimulation (McIntyre et al., 2002; Capogrosso et al., 2013; Marianelli et al., 2015). An electrical field generated by injected current will spread spherically through a space of uniform conductance. Injecting more or less current will change the radius of this sphere. Any afferents within this sphere can be depolarized above the threshold voltage, which will generate an action potential if the cell is not in the refractory period. Resting firing rate statistics were extrapolated based on animal models, including (Fernandez and Goldberg, 1971) squirrel monkeys with 91.3 ± 36.3 spikes/s; (Sadeghi et al., 2007) macaque monkeys population afferent firing rate was approximately 95 spikes/s; (Baird et al., 1988) chinchilla had an afferent population firing rate of 70.9 ± 1.1 spikes/s for regular units, respectively; (Bronte-Stewart and Lisberger, 1994) rhesus monkey had a distribution of resting afferent firing rates from 29 to 158 spikes/s with a peak around 100 spikes/s; and (Hullar et al., 2005) chinchilla population afferent resting firing rate of 42 ± 21.5 spikes/s, However, note that detailed characterizations of this nerve have not been performed in humans or after BVL.

The model will have *N* afferents within the sphere of delivered charge:

(1) $$N=n\;(A_{\text{b}}+A_{\text{m}})$$

where *n* is a ratio between the number of recruited afferents vs. injected current. This weighting is dependent on electrode placement and impedance. Here, we set *n* such that *N* = 40% of afferents (400 afferents out of the 1000 afferents simulated) at baseline (*A*_b_) current while modulated (*A*_m_) stimulation is 0. This reflects the procedure used in patient trials to set the baseline current amplitude in the middle between the threshold current amplitude and the upper comfortable level (Perez Fornos et al., 2014). It is challenging to generalize “typical” values for *A*_b_ and *A*_m_, as the effect of stimulation is dependent on patient anatomy, surgical placement, and electrode condition. Detailed dynamic ranges for a group of 11 patients are given in Guinand et al. (2015). We use 1000 afferents per SCC for this paper. Results are consistent, simulating different numbers of afferents; only runtime and resolution are affected (unpublished).

**Figure 1:**
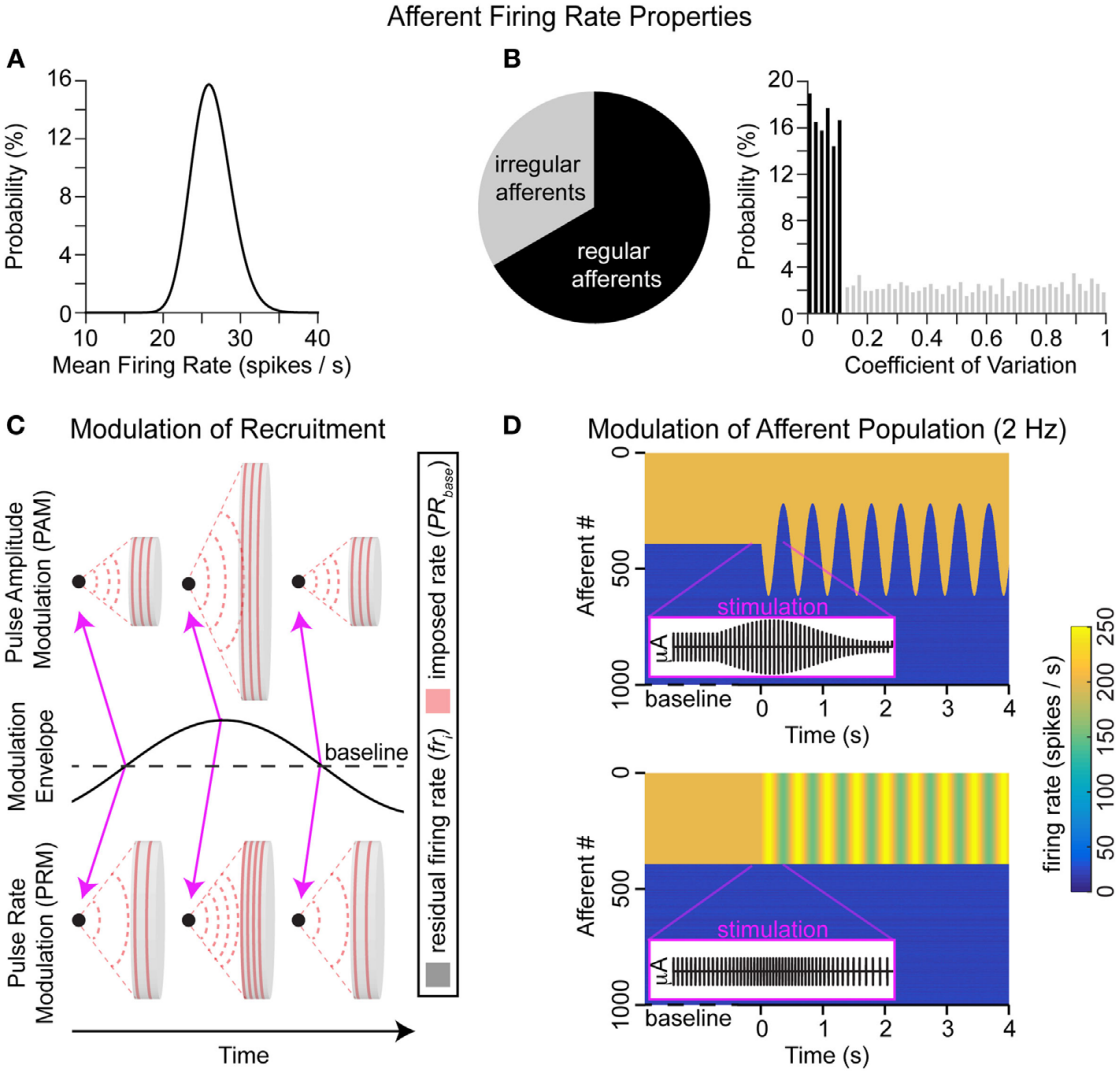
**Electrode–nerve interface**. **(A)** Distributions of resting mean firing rates for the population. **(B)** The percentage of regular (67%) and irregular (33%) afferents is illustrated in the pie plot; the distribution of CVs for each afferent type is also shown. Each afferent will have a mean residual firing rate sampled from **(A)** and a variance sampled from **(B)**. **(C)** Concept of stimulation from a monopolar electrode (black dot) in an environment with uniform conductance. The area stimulated depends on pulse amplitude, while the frequency of action potentials depends on pulse rate. A sinusoidal envelope modulates both modalities from baseline to ± maximum values. This modulation envelope is a 2-Hz sine wave. At baseline, there is no modulation (e.g., *A*_m_ and/or PR_m_ go to 0). Positive (negative) values of the modulation envelope correspond to delivering higher (lower) values of pulse amplitude and/or pulse rate than baseline (e.g., *A*_m_ and/or PR_m_ are non-zero). Pink arrows show that the first and last examples here are at baseline stimulation, while the middle example is a peak positive modulation. **(D)** Simulated vestibular afferent populations during baseline stimulation followed by PAM or PRM. In purple, we overlay the zoom into brief section of the applied stimulation pulses, including baseline, maximum, and minimum modulation values.

We assume that afferents continuously generate action potentials, according to probability distributions of firing rates. Multiple distributions were tested, but for this paper, all mean firing rates were sampled from the lognormal distribution (mean = 26.3 spikes/s and SD = 6.6 spikes/s), as shown in Figure 1A. Each afferent also had a variance in firing rate, depending on whether it was irregular [33% of afferents, coefficient of variation (CV) >0.1] or regular (67% of afferents, CV <0.1), these variances were sampled from the uniform distribution for each type shown in Figure 1B. The percentages of regular and irregular afferents (and CV ranges for each type) were extrapolated based on animal models, including (Fernandez and Goldberg, 1971) squirrel monkeys that had a 1:2 ratio of regular (CV <0.058) to non-regular (CV >0.238) afferents; (Sadeghi et al., 2007) macaque monkeys that had a 3:2 ratio of regular (CV* <0.15) to irregular (CV* >0.15) afferents; (Baird et al., 1988) chinchilla that had 3:1 ratio of regular (CV* <0.1) to irregular (CV* >0.2) afferents; (Bronte-Stewart and Lisberger, 1994) rhesus monkey that had a nearly 6:1 ratio of regular (CV* <0.1) to irregular (CV* >0.2) afferents; and (Hullar et al., 2005) chinchilla had a 1:3 ratio of regular (CV* <0.1) to irregular (CV* >0.2) afferents. For the sub ensemble of *N* afferents influenced by the electrical stimulation, the firing rate is locked to the stimulation rate if it exceeds the residual resting firing rate.

(2) $${\mathrm{fr}}_{\text{i}}=\max\left({\text{PR}}_{\text{b}}+{\text{PR}}_{\text{m}},p(s_{\text{i}},u_{\text{i}})\right)$$

where PR_b_ and PR_m_ are pulse rates of baseline and modulated stimulation, *i* is the afferent index, and *u* and *s* are the mean and variances of the residual resting firing rates, respectively. The difference between the stimulation modalities (PAM and PRM; Figure 1C) that influence on vestibular nerve activity (Figure 1D) is captured with *A*_m_ and PR_m_ in Eqs 1 and 2.

### Nerve–Nuclei Interface

The vestibular nuclei are innervated by multiple components of the nervous system, one of which is partially composed of the afferents within the vestibular nerve. Each afferent synapses onto the nuclei, with the influence of delivered action potentials determined by the synaptic strength (Arnold and Robinson, 1997). Afferents within this vestibular nerve synapse directly onto position-vestibular-pause neurons (Cullen and McCrea, 1993; Sadeghi et al., 2009, 2011) within the vestibular nuclei in the brainstem. Each nucleus receives bilateral input from afferents within the vestibular nerves. We model the strengths of these synapses with linear weight terms. The nuclei sum the weighted firing rates from all afferents and then apply a saturating non-linearity, which encapsulates both non-linearity in the nucleus and in eye muscle contractions [for a detailed model of the individual non-linear elements, see Arnold and Robinson (1997)]. The synaptic strengths are plastic, i.e., they adapt to minimize retinal slip. Prior research has shown that adaptation similar to long-term depression (LTD) (Markram et al., 1997) may account for reduced sensitivity to unilateral afferent inputs at supra-physiological rates (Mitchell et al., 2014).

**Figure 2:**
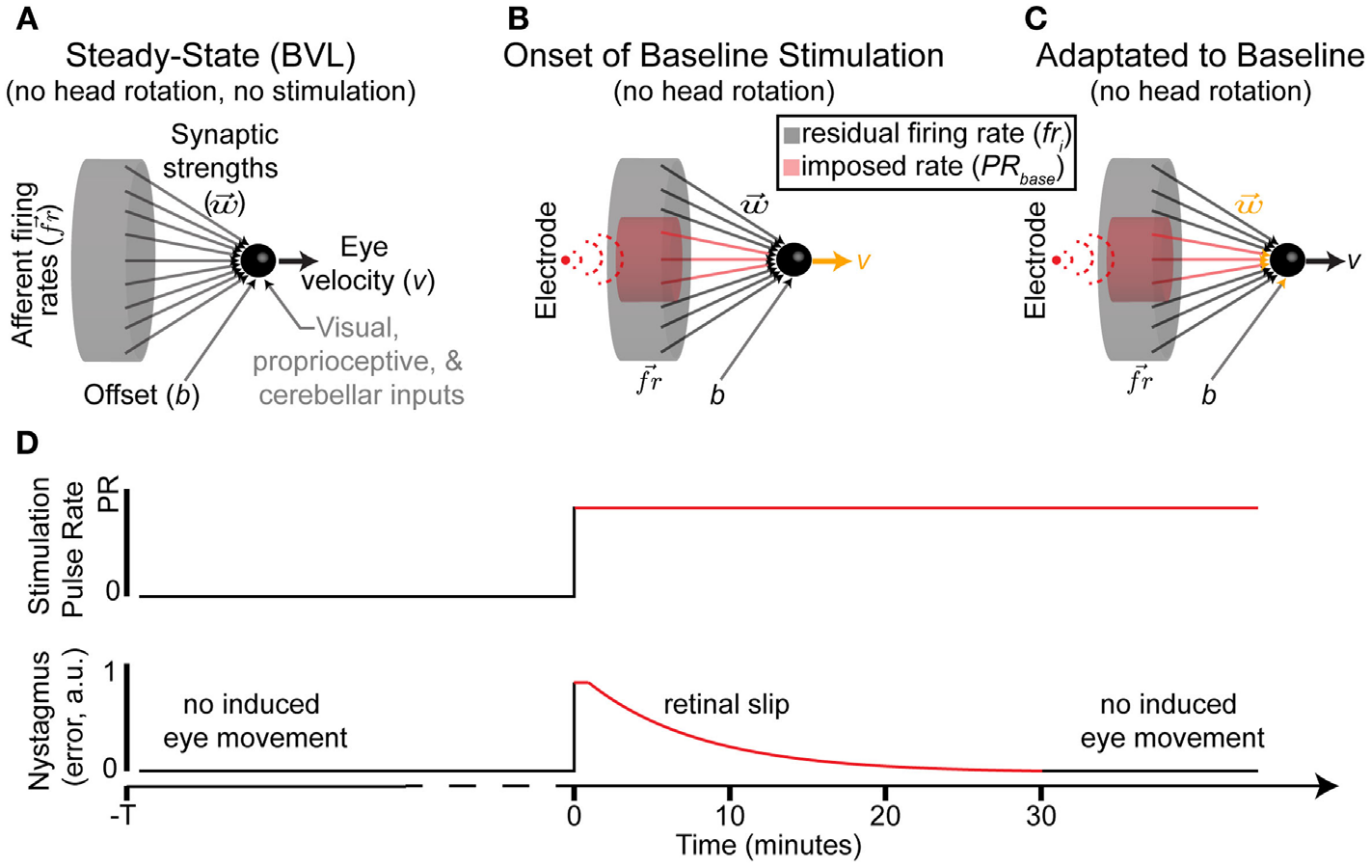
**Nerve–nucleus interface**. **(A)** Model of primary afferents in BVL without prosthetic stimulation. There is no eye movement despite residual afferent activity. **(B,C)** The onset of baseline stimulation introduces imbalances between the synaptic strengths, self-regulation, and induced firing rates within stimulated afferents. Any changes are color coded in orange. **(D)** Nystagmus occurs immediately after baseline stimulation onset, but attenuated in all tested patients within a maximum of 30 min. We model this as a change in synaptic strengths and/or self-regulation in **(C)**.

The final output of these nuclei is an eye velocity command (Figure 2A), which will pass through the abducens nucleus, trochlear nucleus, oculomotor nuclei, and the ocular muscles (Purves et al., 2004). We make three additional assumptions to model these nuclei: (a) inputs (represented as weighted firing rates) from each synapse are summed, (b) an “offset” that can globally attenuate inputs (e.g., rely on vision for slow movement in good lighting), and (c) other inputs are grouped into a single term *P*. Thus, the equation for the model is

(3) $$v=f\left(\sum_{\text{i}}w_{\text{i}}{\mathrm{fr}}_{\text{i}}-b,P\right)$$

where *v* is the observed eye velocity (around a single axis), *w*_i_ is the synaptic strength for each afferent, *i*, *b* is the offset, and *P* represents all other inputs to the vestibular nuclei. These inputs are known to influence gains of the VOR, but the exact methods for achieving this are disputed (Loeb and Tsianos, 2015). For the remainder of this work, we assume *P* is independent of stimulation modality (PRM or PAM) and can be removed from the model, as it will cancel out in any differential comparisons. A non-linear hyperbolic tangent function was used for the mapping *f*() between the nuclei and eye movements; it was selected based on the relationship between firing rate and eye movement previously detailed in monkeys (Fernandez and Goldberg, 1971). This mapping and the removal of *P* reduces Eq. 3 to a standard single-layer perceptron, as described in Haykin (1994).

### Acute Adaptation

After vestibular injury or loss, the vestibular nerve does not provide useful information. The hair cells that normally modulate afferent firing either die or become non-responsive to rotational stimulation. Thus, the population of afferents is no longer modulated by the hair cells and instead reverts to a residual resting discharge that is independent of head rotation. The vestibular nuclei must attenuate the influence of afferent’s residual resting discharge, which may be lower than the healthy resting discharge rate, to minimize retinal slip. Retinal slip (*e* in Eq. 4) is the difference between the appropriate (i.e., perfectly compensating for head rotations) and actual eye movements; this slip induces aberrant percepts of image motion. Experimentally, nystagmus (an eye movement consisting of a slow movement in one direction followed by a rapid movement in the opposite direction, conceptually a series of retinal slips) induced by abrupt onsets of electrical stimulation attenuated to negligible values within a maximum of 30 min while the patient was sitting in a dark room (Guyot et al., 2011). To achieve this attenuation in the model is possible *via* Eq. 5.

(4) $$e=v_{\text{eye}}+v_{\text{head}}$$

(5) $$\begin{array}{l} 0=v_{\text{eye}}\\ 0=f\left(\sum_{\text{i}}w_{\text{i}}{\mathrm{fr}}_{\text{i}}-b\right) \end{array}$$

Setting the sum of weighted firing rates equal to the offset will minimize error. We do not reintroduce *P* from Eq. 3, as we assumed that within the timescale of adaptation to baseline stimulation onset, while the patient is seated in a dark room, the contribution of *P* is constant. Thus, nuclei adaptation is restricted to *w* and *b* (Figures 2B,C).

Mean squared error is back propagated, according to Eqs 6 and 7 (Eq. 7 includes a Fahlman constant; is the error, back propagated through the output node), where *d*_v_ is the desired eye velocity, i.e., opposite of head movement to keep the gaze focused. The synaptic strengths, here represented as a vector, and offset are updated with Eqs 8 and 9.

(6) $$e=d_{\text{v}}-v$$

(7) $$\delta =e\;\times \;(1.1-v^{2})$$

(8) $$\Delta w=\alpha_{\text{s}}\delta \mathrm{fr}$$

(9) $$\Delta b=\alpha_{\text{b}}\delta$$

Here, there are different learning rates for the synaptic strengths (α_s_) and offset term (α_b_). This feature was added to address the uncertainty of the nuclei attenuating all afferent input (α_b_ ≫ α_s_), attenuating only the stimulated afferents (α_s_ ≫ α_b_), or a hybrid approach in response to the discontinuity introduced by the onset of baseline stimulation. This spectrum of relative learning rates was chosen to emulate heterosynaptic and homosynaptic LTD, respectively. Homosynaptic LTD is a Hebbian-type learning that decreases the synaptic strength if both the pre- and postsynaptic neurons are active (Purves et al., 2004). In contrast, heterosynaptic LTD attenuates synapses independently of neuron activity.

### Modulation

**Figure 3:**
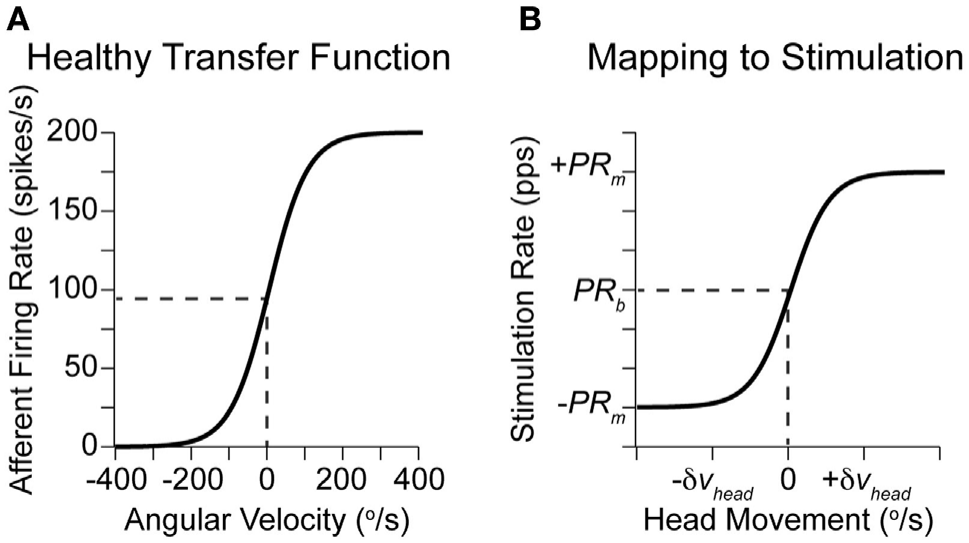
**Relationship between head rotation and afferent activity**. **(A)** Approximate transfer function between head rotations along a single axis and afferent firing rates in healthy monkeys (Fernandez and Goldberg, 1971). **(B)** Imposed transfer function for each axis in a vestibular prosthetic using PRM. The width of the linear region can be set based on the observed patient-specific head rotations or the maximal range of possible rotations (Gong and Merfeld, 2000; Della Santina et al., 2007).

The prosthetic modulates eye velocity commands (Eq. 3), based on the changing afferent firing rates, as described by Eqs 1 and 2. The relationship between actual head rotations and afferent firing rates in healthy monkeys (Fernandez and Goldberg, 1971) is shown in Figure 3A. Similarly, the mapping between detected rotations and prosthetic stimulation is given in Figure 3B for PRM. Equivalently, PAM can be achieved by swapping PR_m_ with *A*_m_.

**Figure 4:**
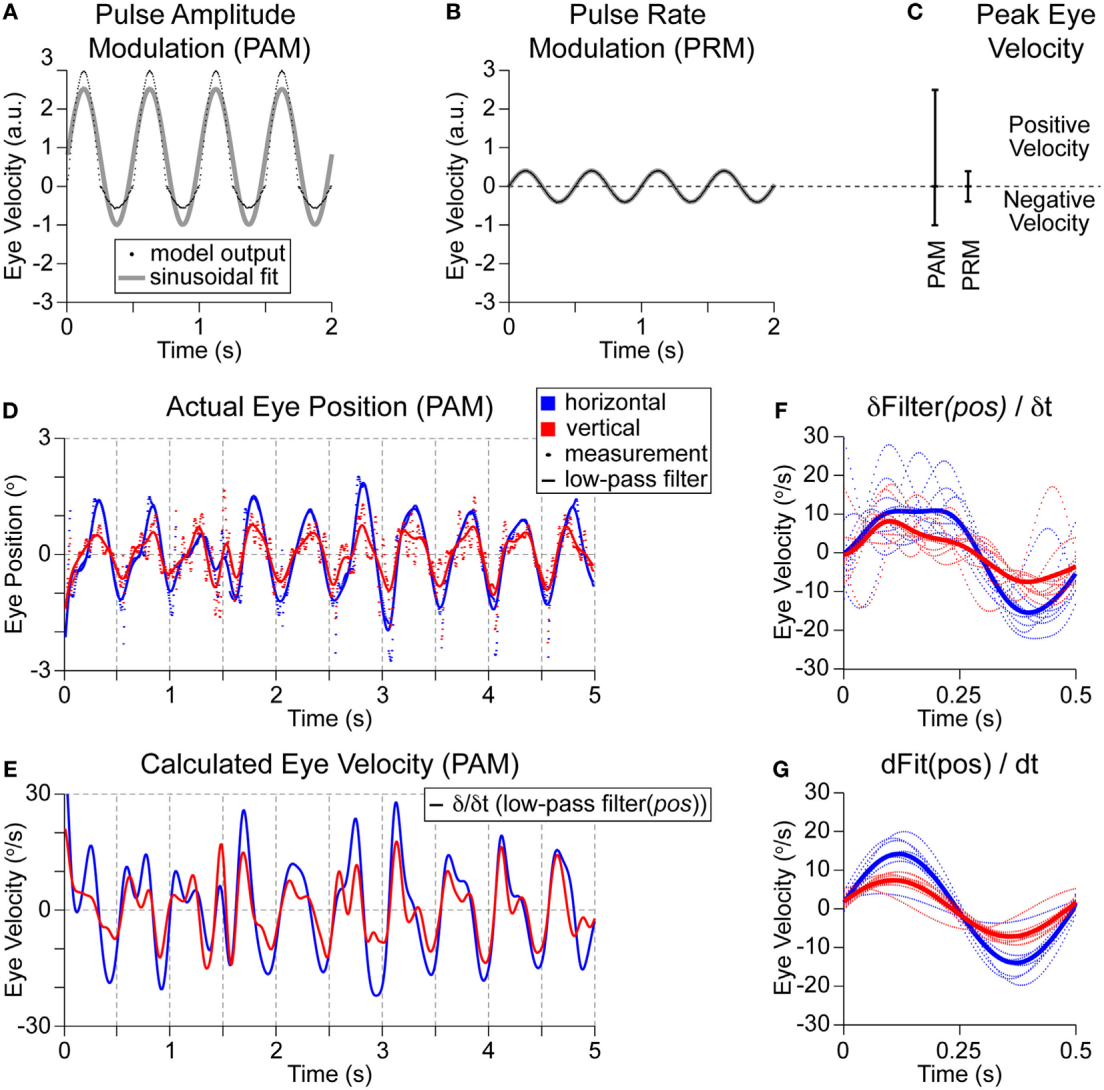
**Model outputs and patient measurements**. Models were trained using clinical settings of 200-pps baseline stimulation and then ±25% modulation depth was applied. **(A)** PAM generated relatively large positive eye velocities with muted negative eye velocities. Applying a sinusoidal fit shows a peak positive eye velocity of 2.5 in arbitrary units. **(B)** Applying PRM to the same model generates much smaller eye velocities, but without any imbalance in positive and negative outputs. There is complete overlap of the model outputs on the sinusoidal fit. **(C)** The peak eye velocities for each modality are shown. **(D)** Representative PAM recording from a single patient during stimulation of the lateral ampullary nerve (which should elicit purely horizontal eye velocities) shows a 2-Hz modulation of eye position (dots) in the horizontal (red) and vertical (blue) dimensions. Traces are generated with a 5-Hz low-pass filter. **(E)** Differentiating these traces estimates eye velocity. **(F)** Compiling all cycles of modulation reveals a broad and uneven positive velocity peak followed by a more sinusoidal shaped and larger magnitude negative velocity peak. **(G)** Alternatively, each cycle of the position signal in **(D)** is fitted with a sinusoid, then differentiated. This dramatically cleans up the velocity outputs but obscures any imbalance in positive and negative outputs or modulation shapes.

For this paper, we apply only a fixed sinusoidal (2 Hz) modulation of stimulation parameters (see Figure 1B) between *A*_b_ + *A*_m_ and *A*_b_ − *A*_m_ independently of any actual head rotations of the vestibular prosthetic user (or between PR_b_ + PR_m_ and PR_b_ − PR_m_). This modulation frequency (2 Hz) was selected because it is a prevalent head frequency in everyday tasks, such as walking (Grossman et al., 1989; Guinand et al., 2015). The desired outcome is to evoke maximal eye velocity. This configuration mimics the acute clinical testing of a prosthetic as shown by Guyot et al. (2010). Outputs for PAM and PRM using common clinical settings are shown in Figures 4A,B. The metric for evaluating performance is the positive peak eye velocity (PEV) shown in Figure 4C. Using only positive, PEV hides the imbalance between positive and negative eye velocities in this model with PAM (Figures 4A,C). However, real eye-movement data (Figure 4D) is noisy, particularly in the measured eye positions during the negative portion of the cycle. Differentiating this signal to calculate velocity compounds the problem, so we first applied a 5-Hz low-pass filter (third order, non-causal Butterworth) to the position (Figure 4D). This creates the expected periodic modulation in the velocity signals, in this example, it is obvious for times >2 s (Figure 4E). However, compiling and averaging these modulation cycles reveals a sinusoid with different phase amplitudes and frequencies (Figure 4F). Alternatively, eye position can be fit for each cycle, as in Perez Fornos et al. (2014), and then differentiated (Figure 4G). This generates a much cleaner output, but the necessary detail (positive vs. negative velocity) for model fitting is lost. Given the noise in the eye velocity calculations (Figure 4F) and the dependency on the cut-off frequency of the low-pass filter, we only consider one portion (PEV) of the model output.

These representative eye positions also highlight another challenge for vestibular prosthetics. In this patient, the lateral SCC was stimulated, which should have evoked purely horizontal eye movements. Instead, there are both horizontal and vertical eye movements. This is an example of the common phenomena of current spread; here, the unwanted movements (vertical) are nearly two thirds as large as the desired (horizontal) movements. Using PAM accesses the horizontal SCC as desired but also spreads to stimulate the anterior SCC (and potentially other unintended targets), which generates unwanted eye movements. Current spread may be reduced by changing the maximal change in PAM; however, this will also reduce the desired horizontal movement magnitude. In the model, the maximal change in stimulation for PRM and PAM was balanced for any comparisons. Specifically, the multiplier from *A*_b_ to *A*_m_ was the same as from *P*_b_ to *P*_m_. We labeled this “charge-balanced.” This multiplier was often 1.25 (or 25%), which has been a common upper bound in current acute clinical PAM testing.

Hybrid comodulation of stimulation was also modeled. All three stimulation modes are summarized by the change in charge in Eq. 10. (Note that the *A*_m_ component also has a geometric multiplier based on Eq. 1. We also removed the pulse-width term, as this is common over methods). In PAM or PRM, only the first or second terms, respectively, are non-zero. In contrast, for hybrid stimulation all three terms are non-zero. We do not restrict hybrid stimulation to be charge-balanced with PAM or PRM. Instead, we characterize possible hybrid model outputs across possible percentages of PRM and PAM.

(10) $$\Delta Q={\text{PR}}_{\text{b}}A_{\text{m}}+{\text{PR}}_{\text{m}}A_{\text{b}}+{\text{PR}}_{\text{m}}A_{\text{m}}$$

## Results

This simple model of vestibular prosthetic and vestibular–ocular interaction exposed two important relationships. First, there may be a large discontinuity between the induced pulse rate and the residual resting discharge of the population of non-recruited afferents. This discontinuity is exacerbated, as higher baseline pulse rates are used. Second, the adaptation to baseline stimulation tends to attenuate the weight of the recruited afferents (e.g., afferents #1–#400 in Figure 1C). Both of these relationships disproportionally degrade the efficacy of PRM.

### Influence of Baseline Pulse Rate and Residual Resting Discharge

**Figure 5:**
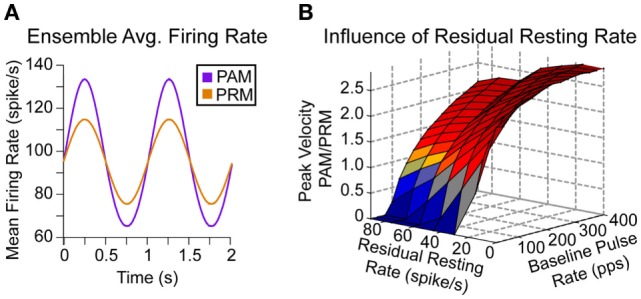
**Ensemble and residual firing rates**. **(A)** Given changes in pulse rate or pulse amplitude, there is a larger modulation in firing rate for PAM. Here, the baseline recruitment (*A*_b_) is fixed to 40% of afferents, modulation (pulse rate or pulse amplitude) is fixed to 25%, and we scan over all residual firing rates and baseline pulse rates. **(B)** There is higher peak eye velocity for PAM (red) over a large region of this space. However, PRM does generate higher peak eye velocities (blue) for low baseline pulse rates, especially if the residual firing rate is also low.

The most influential relationship exposed by this model is the discontinuity between baseline pulse rate and residual resting discharge. In Figure 5A, we compare the average firing rate within the ensemble of afferents for PAM (22.5–62.5% afferents stimulated, 200 pps) and PRM (40% afferents stimulated, 200 ± 50 pps) at 25% modulation for both methods. Note that this average includes many non-modulated afferents (for either PAM or PRM), which lowers the value toward the residual resting firing rates as shown in Figure 1A. For example, afferent #500 (Figure 1C) switches from a mean firing rate of approximately 27 to 200 pps during the positive modulation half-cycle of PAM. The subpopulation of neurons with this discontinuity contributes to the sharp increase in ensemble firing rate. This dependency is described in Eqs 10 and 11, where Δ*fr* is the change in the afferent ensemble firing rate, and all other variables are from Eqs 1 and 2.

(11) $$\Delta {\mathrm{fr}}_{\text{PAM}}=\sum_{i=nA_{\text{b}}+1}^{n(A_{\text{m}}+A_{\text{b}})}{\text{PR}}_{\text{b}}-{\mathrm{fr}}_{\text{i}}$$

(12) $$\Delta {\mathrm{fr}}_{\text{PRM}}=nA_{\text{b}}({\text{PR}}_{\text{m}}-{\text{PR}}_{\text{b}})$$

The baseline population (*n* × *A*_b_) of stimulated neurons subjected to the modulated pulse rate (Eq. 12) is larger than the modulated population [*n* × (*A*_m_ *− A*_b_)], i.e., afferents stimulated only during the positive sine wave of PAM. However, the discrepancy between baseline pulse rate and residual resting discharge in Eq. 11 during the positive sine wave of PAM causes a larger overall change in ensemble firing rate.

In the first acute tests with proof of concept prosthetics (Perez Fornos et al., 2014), 25% modulation depth (i.e., stimulation intensity ranged from 25 to 75% of the dynamic range, where 100% is the upper comfortable level) corresponded to a typical clinical case. Fixing the modulation depth of PR to ±25% of baseline PR, it is clear that PAM will generate eye movements with greater magnitude throughout a majority of possible combinations (Figure 5B).

### Influence of Stimulated Afferents and Learning Rates

**Figure 6:**
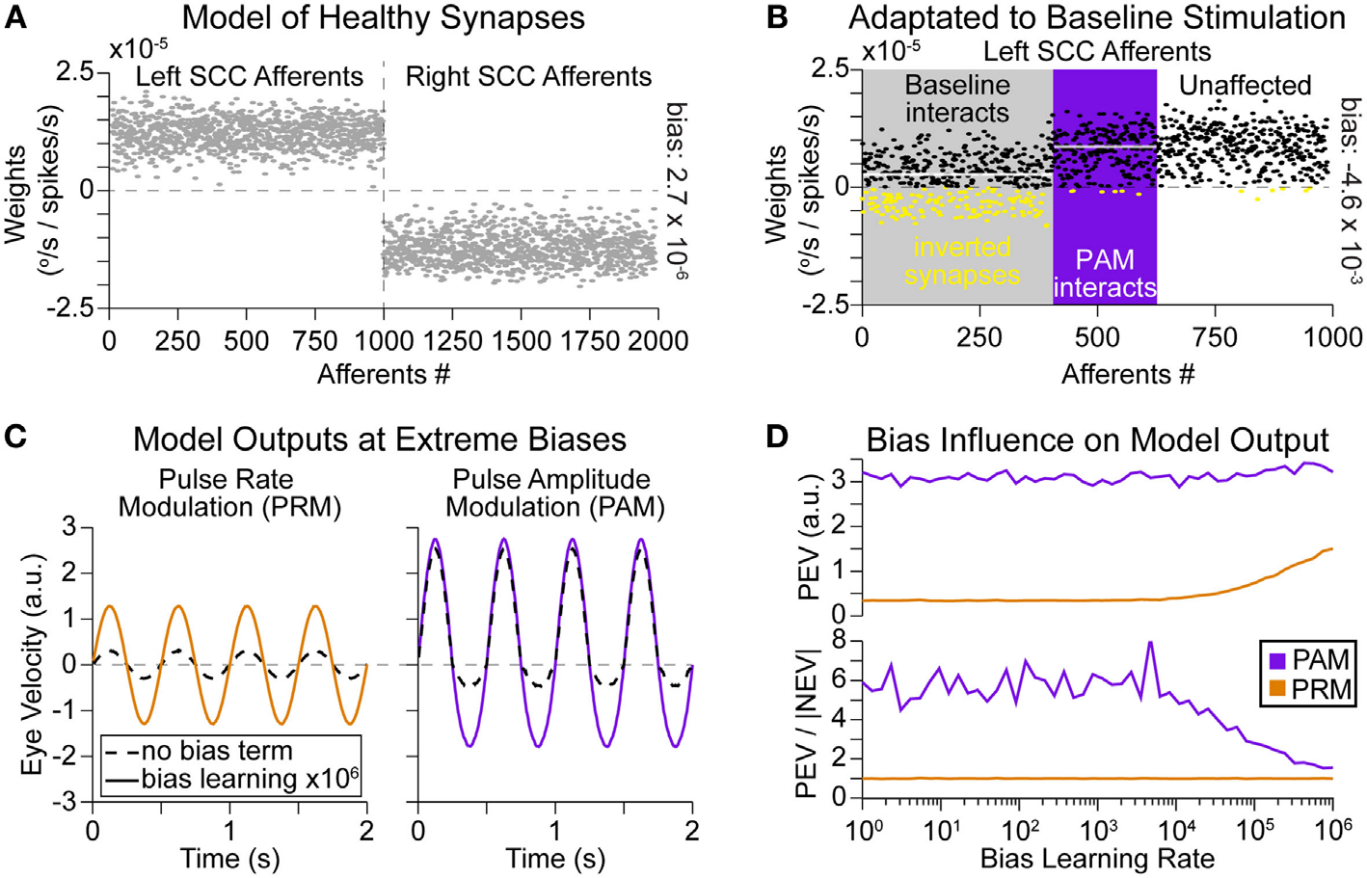
**Synaptic strengths and learning rate ratios**. **(A)** Modeled healthy afferent strength values connecting to the vestibular nuclei. Increased firing rates from left SCC afferents cause positive eye movement (move to right); decreased firing rates from right SCC afferents have the same effect as they are multiplied by a negative strength **(B)** During baseline adaptation to left SCC stimulation by a vestibular implant, there is a population (shown in gray) of afferents entrained to 200-pps activity. This excess activity causes nystagmus (error). To correct this error, the network increases the bias term and adapts the other strengths. Here, the bias strength was 2 × 10^3^. Now, there are strengths in the incorrect region of space (inverted synapses are colored yellow), and increasing firing rates in these afferents would yield negative eye movement. Additionally, the mean strength value is lower in the gray area compared to the rest of the population (mean values shown as white overlay lines for each region). This creates an advantage for PAM, which interacts with a subpopulation of afferents (shown in purple) with larger strengths during the positive phase of modulation. **(C)** The ratio of learning rates between the bias term and neurons is adapted to simulate heterosynaptic (bias rate very high) vs. homosynaptic (synapse rate very high) LTD. The ratio of learning rates affects the magnitude of PRM and the symmetry of PAM. Relatively faster bias term adaptation increases both the factors. **(D)** Simulation of different bias rates showing the increase in PEV for PRM, as bias learning rate increased above 3 × 10^4^. The increase in symmetry (PEV/|NEV| goes toward 1.0) for PAM occurs around the same learning rate.

The differences in outputs can be understood through the synaptic strengths in the model. In the healthy situation, synapses are organized such that increasing firing rates in afferents from a SCC on one side are weighted positively while inputs from the opposite SCC are weighted negatively (Figure 6A). Conceptually, one could consider positive synaptic strengths excitatory while negative strengths are inhibitory. Alternatively, positive and negative may be defined relative to the direction that the eyes are moved. Either interpretation combines the push–pull mechanism of vestibular sensory function to create a single eye movement in the correct direction. After BVL, there is no more natural modulation in afferents from either SCC; instead, all afferents fall to a distribution of residual resting rates (Figure 1A). The synapses in the model are all evenly adapted to accommodate this lower input rate. Later, a unilateral prosthetic is implanted and afferents from a single canal are stimulated tonically at a baseline frequency for approximately 30 min (Figure 2D). We assumed that 40% of afferents would be recruited at this current amplitude. This means 400 afferents (shown in gray in Figure 6B) suddenly went from a low residual firing rate (normally distributed around approximately 27 pps) to the baseline stimulation rate of 200 pps. The model again adapts the weights, according to Eqs 8 and 9; however, there is a sharp difference in the firing rate for this subpopulation of afferents. Thus, synapses for these afferents are adapted more rapidly than the non-stimulated afferents. Figure 6B shows the impacts of this adaptation, many synapses within the recruited population (in gray) become negative and overall the mean value of these synapses is lower than other afferents. This means that increases in firing rate within certain afferents in the recruited population actually contribute to attenuate positive eye movements. It also highlights the subpopulation of afferents (in purple) only influenced during PAM, which have a higher mean synaptic strength. Given the same change in firing rate between two afferents, the afferent with the higher synaptic strength contributes more to generate positive eye movement. The different changes in ensemble firing rates (inputs) and synaptic strengths combine to generate a much higher velocity from PAM than from PRM (Figures 4A,B and 6C).

The ratio of bias vs. synaptic learning rates also affects the model output. At extreme values of bias learning rate (heterosynaptic, solid lines in Figure 6C), the magnitude of model output for PRM increases, and the imbalance between PEV and negative peak eye velocity (NEV) for PAM decreases. Both of these effects are due to adaptation of the bias term completely offsetting any errors at high learning rates. That strategy preserves the afferent weights at nearly the original values (Figure 6A), which boosts PRM output. Not adapting the afferent weights also avoids the discontinuity between baseline and PAM stimulated afferents (Figure 6B); this correspondingly reduces PEV vs. NEV imbalance for PAM. Scanning over possible learning rate ratios in Figure 6D shows that these positive changes occur at bias learning rates that are 3 × 10^4^ times larger than the learning rate for a synapse (or 30 times larger than the combined change in all afferents). Such rapid bias learning rates mitigate the problematic synaptic properties shown in Figure 6B for lower bias learning rates.

### Modulation Efficacy

**Figure 7:**
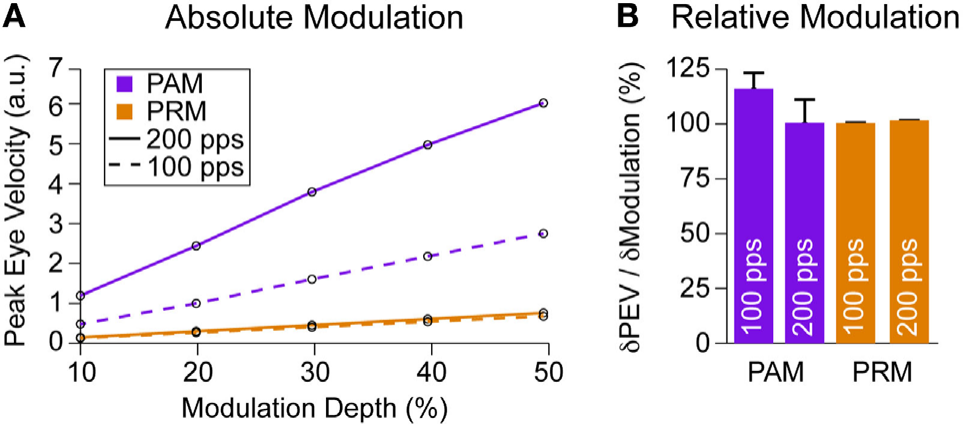
**Modulation efficacy**. **(A)** Peak eye velocity over different modulation strengths for PAM and PRM at 100- and 200-pps baseline stimulation. There is a clear attenuation of PAM at lower baseline stimulation while PRM remains unaffected. **(B)** Normalizing to the change in output at 10% modulation, we see both PAM and PRM increase PEV about 100% per 10% change in modulation.

Precise modulation of eye velocity is important for vestibular prosthetic function. All prior sections focused on peak output using a 200-pps baseline with ±25% modulation depth. We also checked other modulation strengths from 10 to 50%. For two baseline pulse rates, 100 and 200 pps, PAM generated larger eye velocities (Figure 7A). However, we see a large drop in PAM output at the 100-pps baseline due to the PR_b_ term in Eq. 11. This change in baseline does not affect PRM. Normalizing by eye velocity at 10% modulation depth, we see that both PAM and PRM created an approximately unity increase in eye velocity for each additional 10% change in modulation (Figure 7B), indicating similar modulation efficacy.

**Figure 8:**
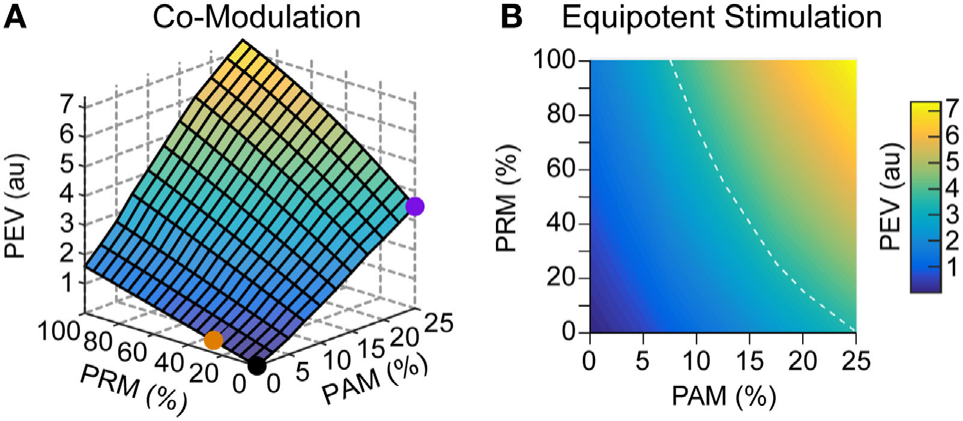
**Comodulation**. An alternative stimulation strategy to PAM and PRM is to modulate both the pulse amplitude and rate of stimulation. **(A)** The orange and purple dots represent pure PAM and PRM, respectively, as previously presented. Combining these modulation methods further increases the possible PEV output. **(B)** Similarly, there are multiple combinations of stimulation methods which are equivalent to 25% PAM. For example, the white dashed line shows that the same output can be achieved with 12.5% PAM combined with 55% PRM.

Finally, we combined the two methods to simulate comodulation. Specifically, both *A*_m_ and PR_m_ were varied together at a 200-pps baseline stimulation rate and 2 × 10^3^ bias learning rate ratio. Adding PRM on top of PAM creates a non-linear multiplier (see Eq. 10) on the eye velocity output (Figure 8A). For example, adding 100% PRM to 25% PAM increases the model output by a factor of 2. Alternatively, we assume that eye velocity at 25% modulation depth for PAM is sufficient for prosthetic function. To reduce current spread and avoid reaching the upper level, there are equipotent contours in hybrid stimulation space (Figure 8B) that can achieve the same eye velocity output with less PAM.

## Discussion

We have presented a simple, functional model of the interaction between vestibular prosthetic stimulation and induced eye velocity. The novelty of this model is the adaptation of vestibular nuclei synaptic strengths to emulate neural plasticity in the processing of afferent activity. We specifically focus on the tonic baseline stimulation period of vestibular prosthetic onset, which initially causes a nystagmus that attenuates after 30 min (Guyot et al., 2011). Since there has been no change in the input, we focused on the synapses to explain the change in the output. LTD was proposed as a physiological mechanism for synaptic adaptation, including both hetero- (all synapses are adapted independently of activity) and homo- [only active (stimulated) synapses are adapted] synaptic components. Spike-time-dependent plasticity, which LTD is a subset of, appears rapid enough to allow such error attenuation. It has been shown to occur with approximately 100 action potential pairings repeated at fixed frequencies (Markram et al., 1997; Bi and Poo, 1998; Sjöström et al., 2001). There is no consensus of the optimal baseline prosthetic stimulation rate; however, even 100 pps already delivers an abundance of action potentials to the stimulated afferents. This adaptation was modeled as back propagation of error with a bias term serving to model homosynaptic components. Specifically, adaptation of the bias term has a relative impact on the contribution of all synapses, regardless of their activity level.

Importantly, we did not focus on model fitting to clinical data. Such patient-specific models have been evaluated (unpublished), but the data were insufficient to test for robust generalization. To date, there are only 12 total patients implanted with some variant of a 1- to 3-branch vestibular prosthetic (Guinand et al., 2015). This makes it impossible to pool data across patients to train the models. Further, eye velocity data are extremely noisy (see Figures 4D,E) (Perez Fornos et al., 2014), and only the positive half of the modulation cycle is reliably present in the recordings. Without a measurement of the PAM output imbalance, there is no reference point to set the ratio of bias to synaptic learning rates beside a very wide range of relative differences in PEV in Figure 6D. There were also modulation frequency dependent gains (Van De Berg et al., 2015) and current spread during PAM to adjacent vestibular structures that caused eye velocities along unwanted axes. These were not addressed in this paper but further diluted the available data. Despite these strong caveats, we were able to reproduce the relative differences in PEV for PAM and PRM at 2 Hz for a 200-pps baseline stimulation as shown in Nguyen et al. (2016)^1^ across a spectrum of residual firing rates and learning rate ratios. We also reproduced the drop in PAM output for 100-pps baseline stimulation without any significant change in PRM output^1^. Interestingly, our model predicts that the same *normalized* modulation is possible using either PAM or PRM. However, since PRM is modulating relative to such a small absolute output, it may be obscured by measurement noise.

This simplified model not only replicated the higher efficacy of PAM of electrical stimulation compared to PRM observed in acute clinical testing (Guyot et al., 2011; Pelizzone et al., 2014; Perez Fornos et al., 2014) but also presented two non-intuitive relationships that may explain this phenomenon. First, physics dictate that PAM will cause a larger overall change in afferent ensemble firing rate than PRM, given the same amount of relative modulation. The charge spreads spherically to recruit a larger population of afferents, and there is a discontinuity between these afferents’ residual resting discharge rates and the (usually much higher) baseline simulation rates. Second, the model sharply attenuates the synaptic strengths for the subpopulation of afferents recruited during baseline stimulation to reduce nystagmus. Synapses for non-recruited afferents are less sharply attenuated. PRM only interacts with the subpopulation of afferents with sharply attenuated synaptic strengths. Combining these relationships, it is clear that PRM is less efficient in enacting a change in ensemble afferent activity and further restricted by lower amplitude synapses. Although this model is limited to synaptic strengths, those parameters are sufficient to predict multiple behaviors. Thus, those parameters may serve an important role in the actual circuit (Loeb and Tsianos, 2015).

Finally, we explored the comodulation of PA and PR, because this strategy had shown good results in chinchilla experiments (Davidovics et al., 2012). By incorporating non-zero PAM, any PRM partially accessed the less-attenuated synapses normally solely available to PAM. Adding a PRM component to the stimulation boosts the eye velocity output of a given PAM percentage. One way to think about this is generating larger total eye velocity that is useful for prosthetics. Alternatively, it is important to consider current spread and misalignment of eye-movement responses shown in Figure 4. This is a limiting factor that prevents prosthetic controllers from using high magnitudes of PAM. As amplitude grows too large, the charge spreads to adjacent canals and begins to generate eye velocities along incorrect axes. To avoid this, the model suggests following an equipotent hybrid stimulation contour to find a comodulation that can avoid current spread or user discomfort while maintaining velocity magnitudes.

If more high-quality data become available, it will be valuable to emulate chronic adaptation to vestibular prosthetic use. A chronic model of vestibular prosthetic function is critically missing. Specifically, there are multiple *chronic* animal studies that demonstrate effective control of eye movements using PRM (Gong and Merfeld, 2002; Wall et al., 2003; Della Santina et al., 2007; Merfeld, 2008; Fridman et al., 2010); this was not captured by any possible parameter combination in our *acute* model. Furthermore, prosthetic designers could rapidly scan different stimulation parameter combinations to minimize adaptation time and error, all the way from acute prosthetic onset in the clinic to chronic outside-the-clinic use.

## Author Contributions

JD designed and implemented the model. JD and TAKN revised the model. TAKN, AP-F, and NG performed the clinical recordings. TAKN and AP-F analyzed the data. All authors conceived aspects of the experiments and analysis. JD and TAKN prepared the figures with the help of the other authors. JD and TAKN wrote the manuscript, and all the authors contributed to its editing. SM and AP-F supervised all aspects of the work.

## Conflict of Interest Statement

The authors declare that the research was conducted in the absence of any commercial or financial relationships that could be construed as a potential conflict of interest.

## Footnotes

^1^Nguyen, T. A. K., DiGiovanna, J., Cavuscens, S., Ranieri, M., van de Berg, R., Guinand, N., et al. (2016). Characterization of pulse amplitude and pulse rate modulation for a human vestibular implant during acute electrical stimulation. *J. Neural. Eng*. (under review).
